# Carcass Characteristics and Meat Quality of Karacabey Merino lambs Reared under Triticale and Oat Pastures Compared with Stall-Fed Lambs

**DOI:** 10.3390/ani13213322

**Published:** 2023-10-25

**Authors:** Hulya Hanoglu Oral, Pembe Dilara Kecici, Firat Alaturk, Cemil Tolu, Bulent Ekiz, Ahmet Gokkus

**Affiliations:** 1 Department of Animal Sciences and Technologies, Faculty of Applied Sciences, Muş Alparslan University, 49250 Muş, Turkey; 2 Department of Animal Breeding and Husbandry, Faculty of Veterinary Medicine, Istanbul University—Cerrahpasa, 34500 Istanbul, Turkey; dilara.kecici@iuc.edu.tr (P.D.K.); bekiz@iuc.edu.tr (B.E.); 3 Department of Field Crops, Faculty of Agriculture, Çanakkale Onsekiz Mart University, 17100 Çanakkale, Turkey; alaturkf@comu.edu.tr (F.A.); agokkus@yahoo.com (A.G.); 4 Department of Animal Science, Faculty of Agriculture, Çanakkale Onsekiz Mart University, 17100 Çanakkale, Turkey; cemiltolu@comu.edu.tr

**Keywords:** feeding systems, concentrate fed, pasture lambs, Karacabey Merino

## Abstract

**Simple Summary:**

An economical livestock production model in which all the needs of the lambs can be met and they reach the optimal slaughter weight in the shortest possible time is the primary goal of all production models. Since the production system is an important factor for carcass and meat quality, different production systems have been developed. In intensive systems with concentrates, carcasses tend to be fatter and reach optimal slaughter weight faster, whereas pasture-fed animals have a better fatty acid profile and redder colour due to higher physical activity. In our study, we investigated the effects of feeding system, birth type, gender, and birth year on the carcass and meat quality of Karacabey Merino lambs. The effects of gender and birth type on Karacabey Merino lambs showed the expected results: single-borns have more muscle tissue than multiples, males have more muscle tissue than females, and females have higher carcass fatness. In conclusion, a pasture-based feeding system is more suitable for Karacabey Merino lambs than a stall feeding system, if lean and tender meat is preferred. However, it should not be ignored that lambs fed in stalls showed better fattening performance in terms of conformation and fatness, during the same period.

**Abstract:**

Fifty-eight Karacabey Merino lambs were used to study the effects of feeding system (triticale pasture, oat pasture, or stall-fed), birth type (single or multiple), gender (male or female), and birth year (2016 or 2017) on various carcass and meat quality characteristics. Stall lambs had higher conformation (CS) and fatness (FS) scores, and higher meat L* and h* values than the two pasture groups, possibly due to higher fat content, while oat pasture lambs had the most tender meat. Single-born lambs had higher CS, FS, and Longissimus thoracis muscle section area, while females had higher subcutaneous and non-carcass fat deposits than their counterparts. Both single-born lambs and 2016-born lambs had higher meat a* and C* values than their counterparts. In conclusion, a pasture-based feeding system is more suitable for Karacabey Merino lambs than a stall feeding system, if lean meat and/or meat products are preferred. However, it should not be ignored that stall-fed lambs showed a better fattening performance in terms of conformation and fatness, during the same period.

## 1. Introduction

The traditional model of lamb meat production in Turkey is mostly based on natural pastures, in the form of extensive or semi-intensive systems. In these production systems, lambs are usually raised with their mothers until slaughter to produce high-quality meat at a low cost. However, sheep milk and dairy products are very valuable, and as a result of these production methods, low cheese production and low slaughter weights occur [1,2]. In addition, inadequate pasture lands and nutritional value of pastures do not meet the needs of the animals, and as a result of many problems, intensive rearing has recently become widespread because it is easier to manage. In intensive lamb production, breeders tend to wean lambs at 45 days or 3 months of age, depending on their needs for milk or dairy production, and provide them with high-energy concentrates, usually ad libitum, to shorten the fattening period before slaughter [1].

An economical livestock production model—in which all the needs of the lambs can be met and they can reach the optimal slaughter weight in the shortest possible time—is the primary goal of all production models. Since the production system is an important factor in carcass and meat quality [2,3], numerous feeding models are used to achieve this goal. In intensive or semi-intensive production systems with concentrate feed, carcasses tend to be fatter and reach their optimal slaughter weight in a shorter time [4,5,6], while those fed pasture-based diets have a better fatty acid profile [7,8,9] and a redder colour, due to the increased physical activity during grazing [10,11]. On the other hand, higher carcass yield [5,7,8], marbling, and tenderness [3,12] with lighter meat colour [5,8] can be observed in concentrate-based production systems due to higher fat content. 

Turkey has a remarkable number of sheep (45.18 million heads), which is almost one-third of that in the European Union (126.85 million heads), and indigenous sheep breeds/genotypes account for almost 92% of these sheep [13]. However, in the last century, many crossbreeding studies have been conducted to improve both the live weight and meat quality of indigenous breeds. The Karacabey Merino is one of these breeds that originated from the German Mutton Merino and the Kıvırcık sheep in Karacabey State Farm and is one of the best crosses in Turkey in terms of meat development [14,15]. Therefore, there is a need for a production system that can be used for fast and high-quality meat production.

The aim of this study was to comparatively investigate the effects of feeding system (triticale pasture, oat pasture, or stall feeding), birth type (single, multiple), gender (female, male), and year (2016 or 2017) on some carcass and meat quality characteristics of Karacabey Merino lambs.

## 2. Materials and Methods

### 2.1. Animals and Production Systems

The study was conducted at the Bandırma Sheep Breeding Research Institute between 2015 and 2017. To obtain animal materials for the study, purebred Karacabey Merino ewes were mated with purebred Karacabey Merino rams in August–September 2015 and 2016. Lambs were born in January–February of both years (2016–2017) and birth dates were recorded in detail (date of birth, birth type, gender, birth weight). Twelve ewes were assigned to a feeding group each year. Three subgroup replicates (3 pens containing 4 sheep in each) were created for each feeding type for repetition. All ewes in the study were three years old when they gave birth to the first group of lambs in 2016, which was their second pregnancy. Ewes were assigned to the predetermined feeding groups prior to the birth season, and both ewes and lambs in each study group were randomly selected.

Since the dry matter (DM) production of pasture types is different, parcel sizes were determined according to the yield of the grazing areas to meet the roughage needs of the animals during the grazing period. For this purpose, the maintenance dry matter requirement of the sheep is taken as the basis [16]. Accordingly, two-stage rotational grazing areas (600 + 480 = 1080 m^2^/parcel) were created for each of the three ewe subgroups and their lambs in triticale and oat pastures. After the first area (600 m^2^) was consumed, animals were moved to the second grazing parcel (consisting of 480 m^2^). The ewes grazed freely with their lambs in each parcel. Ewes were not fed additional to the pasture. Open front mobile birthing compartments were placed in each parcel before the birth season. All lambs in the pasture groups were born in these birth lots. Routine mother–newborn care was provided until the age of 10 days. After the age of 10 days, the lambs were placed in pasture plots with their dams. The ewe and lambs were always together, and the lambs suckled their dams freely. The annual pasture yield of the oat pasture was 165.38 kg DM/ha in 2016 and 85.62 kg DM/ha in 2017, and that of the triticale pasture was 122.67 kg DM/ha and 54.28 kg DM/ha in 2016 and 2017, respectively. Mean DM intake of per ewe in pasture plots varied according to year. In the oat pasture, it was 1808.6 g DM/day in 2016 and 1653.2 g DM/day in 2017, while it was 1266.8 g DM/day in 2016 and 1710.6 g DM/day in 2017 for triticale pasture. Grass samples and intake measurements of animals in the pasture were taken at monthly intervals. Two wired cages (2.5 m × 2.5 m) were placed on grazing plots before the animals were released to determine herbage production and intake.

Detailed information about feeding groups in the study is given as follows:

- Triticale pasture-based feeding system (TP): In this system, lambs were born and raised in the winter pasture with their mothers from birth until approximately 120 days of slaughter age (123.72 ± 2.84 days). Ad libitum good quality alfalfa hay and ad libitum concentrate feed (concentrated feed containing 18% crude protein (CP) and 2700 kcal/kg metabolisable energy (ME), consisting of barley, corn, sunflower meal, marble powder, salt, and vitamin–mineral mixture) under creep feeding conditions were given to lambs after 10 days of age in addition to their mother’s milk. Concentrated feed was not given to the lambs from the age of 1 month until slaughter.
- Oat pasture-based feeding system (OP): In this system, lambs were born and raised in the winter pasture with their mothers from birth until approximately 120 days of slaughter age (121.17 ± 2.79 days). Ad libitum good quality alfalfa hay and ad libitum concentrate feed (same concentrate feed was used in all groups) under creep feeding conditions were given to lambs after 10 days of age in addition to their mother’s milk. Similar to TP lambs, concentrated feed was not given to the lambs from the age of 1 month until slaughter. From the age of 1 month to slaughter, they were fed only oat pasture and their mother’s milk.
- Stall group (SG): Lambs in the stall group were reared intensively and had no access to pasture during the study. SG lambs were fed with mother’s milk from birth, and from the age of 10 days, they were divided into groups in small paddocks, and alfalfa hay (15.6% CP and 2160.40 kcal/kg ME) and concentrate feed were provided ad libitum for the transition to creep feeding, in addition to their mother’s milk. The lambs were weaned when they reached the age of two months, and lamb grower pellets containing 16% CP and 2600 kcal/kg ME were given in addition to ad libitum dry vetch grass (13.6% CP and 1410.40 kcal/kg ME) as roughage. The values reported by NRC [17] were taken as a reference for the nutrition of SG. To prevent the formation of urinary stones in lambs, 1% ammonium chloride was added to the lamb grower feed after two months of age. The number of ewes and lambs used in each group is presented in Table 1, and the nutritional content of all pastures used in the study is presented in Table 2.

In the second year of the project, the same feeding programme was applied as in the first year. At approximately 120 days of age, 58 lambs (27 in 2016 vs. 31 in 2017; 33 female vs. 25 male; 31 single vs. 27 multiple born lambs) were slaughtered to compare the carcass and meat quality characteristics according to their feeding systems.

At the beginning of the experiment, the ewes were randomly distributed among the experimental groups. Milk yields were measured during the experiment; however, no detailed evaluation of milk yields was made in this article. In 2016, the mean (mean ± standard error) daily milk yields in the oat, triticale, and stall groups were 0.575 ± 0.08 g, 0.566 ± 0.06 g and 0.487 ± 0.06 g, respectively. In 2017, 1.055 ± 0.09 g, 0.873 ± 0.07 g, and 1.069 ± 0.07 g were determined for daily milk yield in the same order in the groups.

### 2.2. Slaughtering of Lambs and Carcass Characteristics

Lambs were fasted for 12 h before slaughter, with only access to ad libitum water, in accordance with the research institute’s routine pre-slaughter procedures.

The slaughter of the lambs was performed at the Bandırma Sheep Research Institute Slaughterhouse, without any stunning, immediately after the live weights were recorded. Following the bleeding process, the head, skin, feet, and all internal organs were removed. In addition, the full and empty weights of the stomach and intestines, omental and mesenteric fat weights, and weight of gastrointestinal contents were recorded. To eliminate the additional variation that the amount of gastro-intestinal content would create in terms of carcass yield, after the gastro-intestinal contents were removed, the empty body weights were calculated. Immediately after the carcass dressing, the pH0 and carcass temperature were measured between the 12th and 13th thoracic vertebras using a digital pH metre (T-205, Testo SE & Co. KGaA, Titisee-Neustadt, Germany) from the Longissimus thoracis (LT) muscle. The pH metre was calibrated with two different buffer solutions (pH: 4 and pH: 7), before the measuring of the first carcass in each sampling time. However, due to the temperature difference between cold storage and the slaughterhouse’s ambient temperature, the pH metre was calibrated twice (both in room temperature and inside of the cold storage unit) before the first pH measurement for a more successful recording. The hot carcass weights of all carcasses were recorded and transferred to a cold storage unit at 4 °C.

After the carcasses were kept in cold storage for one day, conformation and fatness scores were given according to the EUROP System [18,19], and the scores were converted to a 1–15 scale [20] for statistical analysis. Following scoring, the LT muscle pH_24_ levels were measured from the same point where pH_0_ measurement was performed. Later, cold carcass weights were recorded, and cold carcass yields were calculated using empty body weights. The kidney knob and channel fat (KKCF), kidneys, and tails were separated from the carcasses, and the weights of these pieces were recorded. After all the carcasses were divided into right and left halves from the median line, a section was made between the 13th thoracal and the 1st lumbal vertebrae of the right half of the carcasses, and the cross-sectional area of the LT muscle was drawn with the help of tracing paper. The backfat thickness was then recorded from the same section point with the help of a calliper [21]. The left halves of all carcasses were divided into six parts as the neck, shoulder, ribs (anterior rib + loin), flank, hind limb, and tail using the method described by Colomer-Rocher et al. [22] and the weights of all parts were recorded.

### 2.3. Meat Quality Characteristics

To determine the meat quality characteristics, the Longissimus thoracis et lumborum muscle of the left half of the carcass were used. Meat colour, express juice, drip loss, cooking loss, and Warner Bratzler (WB) shear force measurements were performed 5 days after slaughter in the Istanbul University—Cerrahpaşa Faculty of Veterinary Medicine, Department of Animal Breeding and Husbandry Carcass and Meat Quality Laboratory. Samples were stored at 4 °C until the day of analysis.

For drip loss analysis, approximately 80 g of samples were taken from the LT muscle, weighed both before and after being hung in a polyethylene bag with a rope without touching the bag, and kept at 4 °C for 24 h. The drip losses of the samples were calculated as the percentage of the weight loss relative to the initial weight.

During express juice analysis, the “Modified Grau and Hamm” method of Beriain et al. [23] was applied. For this purpose, a 30-g piece taken from the LT muscle was separated from its fat and nerves, divided into 5–6 pieces with a total weight of 5 g, and placed between two filter papers. A weight of 2.250 kg was placed on the meat between the filter paper for 5 min, and the liquid rate lost by the meat sample was calculated as % by weighing the filter papers used in the analysis at the end.

A chromometer (CR 400, Konica Minolta Inc., Tokyo, Japan) measuring with L*, a*, and b* coordinate system was used, and the standards reported by CIE [24] were applied during the measurements for meat colour analysis. D65 was chosen as the light source, while aperture size and observation angle were set as 8 mm and 2°, respectively. Chroma (C*) and hue (h*) values were calculated with the given formula; C*= [(a*)^2^ + (b*)^2^]^1/2^; h*= tan^−1^(b*/a*); as described by Murray [25]. A sample of 4 cm thickness was taken from M. *longissimus lumborum*, thrice-repeated 3 measurements (total of 9 measurements) were made from the lean parts of the sample’s cross-sectional surface by a chromometer. The average of the obtained values was calculated, and the colour coordinates were accepted as measurement values. Colour analysis of each sample was performed after 1-h blooming. In this process, the samples were stored at 4 °C under continuous white light.

For cooking loss, 6–8 cm long samples taken from LT muscle were weighed before starting the analysis, placed in heat-resistant polyethylene bags, vacuum packed, and cooked in a water bath at 80 °C for 45 min. Afterwards, they were removed from the water bath and cooled under running water for 1 h. Subsequently, the samples were taken out of their bags and dried with paper towels, and their weights were recorded after cooking. Cooking loss (%) was calculated as the percentage of the difference between pre- and post-cooking weights relative to the initial weight. Cooking loss analysis were performed as one cooking batch per year.

In WB shear force analysis, the samples used in the cooking loss analysis were used, and 6 sub-samples of 1 cm × 1 cm cross-section and 3 cm long were taken parallel to the muscle fibres for each sample. A Warner–Bratzler blade connected to an Instron 3343 (Instron Corp., Norwood, IL, USA) device was used to determine the peak shear force. The peak shear force value of the LT muscle of a lamb was determined by averaging the measurements obtained from all samples taken from the muscle.

### 2.4. Statistical Analysis

The General Linear Model (GLM) procedure was applied in the SPSS 21.0 (SPSS, IBM, Armonk, NY, USA) package programme in order to determine the effects of feeding system, birth year, birth type, and gender on slaughter, carcass, and meat quality characteristics of Karacabey Merino lambs. Feeding system (triticale pasture (TP), oat pasture (OP), or stall fed (SG), year (2016 or 2017), birth type (single or multiple), and gender (male or female) factors were included as fixed effects in statistical models for all investigated parameters in the study. All two-way interactions were included in the models for all parameters. However, non-significant interactions were excluded from the models, using stepwise backward elimination. In case of significant interactions, one-way ANOVA was performed using Duncan’s Multiple Range Test to determine which differences between subgroups were significant. In addition, none of the interactions had a significant effect on the percentages of carcass joints (in Table 3); therefore, they were not presented. Approximate F-ratio tests were conducted for all fixed factors for all parameters and *p* < 0.05 were accepted as the critical value threshold. The least significant difference was chosen for adjustment of multiple comparisons. The means and standard errors presented in the tables are the results from the models. The statistical model used in the analysis as follows:

Yijkl: µ + ai + bj + ck + dl + eijklm

Yijkl: Estimation result of any trait
µ: Overall mean value
ai: Fixed effect of feeding system (i: Stall, Triticale, or Oat)
bj: Fixed effect of birth year (j: 2016 or 2017)
ck: Fixed effect of birth type (k: Single or Multiple)
dl: Fixed effect of gender (l: Male or Female)
eijklm: Residual random error.

**Table 3:** Birth weight and certain slaughtering characteristics of lambs according to feeding group, birth type, and gender.

	n	BW, kg	SA, days	SW, kg	EBW, kg	CCW, kg	CS	FS	SL, %	DP, %	*LTMSA*, cm^2^	OMF, %	BT, mm	KKCF, %
**Group**														
Stall	17	5.30 ± 0.15	127.07 ± 3.05	39.87 ± 1.31	33.91 ± 1.11	19.17 ± 0.70	8.39 ^a^ ±0.29	8.16 ^a^ ± 0.25	2.91 ± 0.24	56.93 ± 0.46	16.30 ± 0.84	1.08 ^a^ ± 0.08	4.65 ± 0.48	1.24 ^a^ ± 0.10
Triticale	20	5.15 ± 0.14	123.72 ± 2.84	37.01 ± 1.22	30.49 ± 1.06	17.25 ± 0.65	7.26 ^b^ ±0.28	6.51 ^b^ ± 0.23	2.98 ± 0.22	55.48 ± 0.43	15.81 ± 0.78	0.80 ^b^ ± 0.08	3.11 ± 0.45	0.75 ^b^ ± 0.09
Oat	21	5.26 ± 0.14	121.17 ± 2.79	38.54 ± 1.19	32.37 ± 1.02	18.03 ± 0.64	8.16 ^b^ ± 0.27	6.87 ^b^ ± 0.23	3.13 ± 0.22	55.64 ± 0.42	16.80 ± 0.77	0.87 ^b^ ± 0.07	3.35 ± 0.44	1.20 ^a^ ± 0.09
**Birth Type**														
Single	31	5.65 ± 0.11	124.18 ± 2.30	40.00 ± 1.00	33.94 ± 0.86	19.18 ± 0.53	8.47 ± 0.22	7.68 ± 0.20	2.94 ± 0.18	56.31 ± 0.34	17.53 ± 0.63	0.97 ± 0.07	3.71 ± 0.36	1.13 ± 0.08
Multiple	27	4.82 ± 0.12	123.80 ± 2.46	36.95 ± 1.06	30.57 ± 0.90	17.12 ± 0.57	7.40 ± 0.24	6.69 ± 0.20	3.08 ± 0.19	55.73 ± 0.37	15.08 ± 0.68	0.83 ± 0.07	3.69 ± 0.49	1.00 ± 0.08
**Gender**														
Male	25	5.41 ± 0.13	124.16 ± 2.58	40.32 ± 1.10	33.62 ± 0.94	18.62 ± 0.59	7.37 ± 0.25	6.87 ± 0.22	3.36 ± 0.20	55.20 ± 0.38	16.67 ± 0.71	0.72 ± 0.08	3.17 ± 0.41	0.86 ± 0.08
Female	33	5.07 ± 0.10	123.82 ± 2.19	36.63 ± 0.94	30.90 ± 0.80	17.68 ± 0.50	8.50 ± 0.22	7.51 ± 0.18	2.65 ± 0.18	56.84 ± 0.33	15.94 ± 0.60	1.09 ± 0.07	4.23 ± 0.34	1.27 ± 0.07
**Year**														
2016	27	5.10 ± 0.13	117.89 ± 2.51	39.79 ± 1.08	34.23 ± 0.92	19.44 ± 0.58	8.40 ± 0.24	7.81 ± 0.21	2.79 ± 0.19	55.82 ± 0.39	16.53 ± 0.69	1.02 ± 0.07	3.43 ± 0.40	1.12 ± 0.08
2017	31	5.39 ± 0.11	130.09 ± 2.25	37.16 ± 0.97	30.29 ± 0.83	16.86 ± 0.52	7.48 ± 0.22	6.57 ± 0.18	3.22 ± 0.17	56.22 ± 0.35	16.08 ± 0.62	0.81 ± 0.06	3.96 ± 0.36	1.01 ± 0.07
**Overall mean**		5.24 ± 0.08	127.99 ± 1.66	38.47 ± 0.71	32.26 ± 0.61	18.15 ± 0.38	7.95 ± 0.16	7.19 ± 0.14	3.01 ± 0.13	56.02 ± 0.25	16.31 ± 0.46	0.92 ± 0.04	3.70 ± 0.26	1.06 ± 0.05
**Significance**														
Group		0.744	0.381	0.286	0.093	0.146	**0.015**	**<0.001**	0.786	0.054	0.656	**0.041**	0.056	**0.001**
Birth Type		**<0.001**	0.912	**0.042**	**0.011**	**0.011**	**0.002**	**0.001**	0.598	0.263	**0.012**	0.093	0.962	0.248
Gender		**0.050**	0.922	**0.015**	**0.034**	0.239	**0.002**	**0.029**	**0.014**	**0.002**	0.441	**0.001**	0.059	**0.001**
Year		0.106	0.001	0.079	**0.003**	0.002	**0.007**	**<0.001**	0.106	0.467	0.641	**0.023**	0.330	0.348
GR×GE					**0.027**		**0.027**	**-**						
BT×Y							**0.002**	**-**						
GR×Y							**0.010**	**<0.001**	**0.038**			**<0.001**	**0.002**	**<0.001**
GE×Y							**-**	**0.005**						
BT×GE										**0.003**				

n: Number of animals in a group, BW: Birth weight, SA: Slaughter age, SW: Slaughter weight, EBW: Empty body weight, CCW: Cold carcass weight, CS: Conformation score, FS: Fatness score, SL: Shrinkage loss, DP: Dressing percentage, LTMSA: Longissimus thoracis muscle section area, OMF: Omental mesenteric fat, BT: Backfat thickness, KKCF: Kidney knob and channel fat percentage, GR: Group, BT: Birth type, GE: Gender, Y: Year. The results in the table are presented as least square means ± standard errors. ^a,b^: The difference between the mean values expressed with different letters in the same column for the feeding groups is statistically significant.

## 3. Results

### 3.1. Slaughtering Characteristics

Birth weights and certain slaughtering characteristics of lambs in three different feeding groups are given in Table 3. The effect of the feeding group was significant on both conformation and fatness scores with omental mesenteric fat and KKCF percentages. SG lambs had higher conformation, fatness, and omental mesenteric fat percentages than their counterparts, while TP lambs had lower KKCF percentages than the others. The effect of birth type on birth weight (BW), empty body weight (EBW), cold carcass weight (CCW), conformation score (CS), fatness score (FS), and Longissimus thoracis muscle section area (LTMSA) was significant, and it was determined that single-born lambs had higher values for these characteristics. Although females have lower birth weight, slaughter weight, and empty body weight than males, their carcasses have higher conformation and fatness scores, dressing percentage, omental mesenteric fat, and KKCF percentages. EBW, CS, FS, shrink loss (SL), dressing percentage (DP), omental–mesenteric fat (OMF), backfat thickness (BT), and KKCF were significantly affected by various interactions. Even though the effect of group was not significant on EBW, male SG lambs had higher EBW than their female counterparts and both TP gender groups. However, the difference between them and OP lambs was not significant. Multiple interactions affected the conformation score, resulting in male TP lambs, multiple-born 2017 lambs, and OP lambs born in 2017 having the lowest conformation scores. Group × year and gender × year interactions affected the fatness score. Female TP lambs had the lowest fatness scores, after the female OP lambs, resulting in all male groups and female SG lambs having higher FS than them. In addition, male lambs born in both years were the fattiest, while 2017-born females had the leanest carcasses. Shrinkage loss of both pasture groups (TP and OP) in 2017 was higher than that of SG in 2016, but the difference between the rest of the groups was not significant. Meanwhile, the dressing percentage of single born female lambs was higher than all of their counterparts. The OMF, BT, and KKCF were affected from group × year interaction, and similar to conformation and fatness scores, the internal fat and muscle accumulation of 2017 TP lambs were the lowest. In addition, SG in 2017 had the highest BT, while 2016 OP group had the highest KKCF percentage, except that the difference between 2016 OP and 2017 SG groups was not significant.

When the carcass joints of the lambs were investigated, the effect of the feeding group on the shoulder, flank, anterior rib, and tail percentages was significant (Table 4). Carcasses of TP lambs had a higher shoulder percentage than SG lambs, whereas the difference between OP lambs and the others was not significant. SG lambs had higher mean values compared with TP lambs, in terms of flank and tail percentages. The OP group had higher percentages for anterior ribs compared with the TP group, but the difference between the TP and SG was not significant. However, the effect of birth type was only significant on kidney percentage, and multiple lambs had a higher kidney percentage than single lambs. When the percentages of carcass joints were evaluated in terms of gender, males had higher shoulder and kidney percentages than females, whereas females had higher loin percentages. Year also had a significant effect on carcass joint percentages. Lambs born in 2017 had higher shoulder and kidney percentages, while lambs born in 2016 had higher flank, neck, and tail percentages. However, year did not affect the first category cuts (*p* < 0.05) In addition, none of the interactions affected the percentages of carcass joints significantly.

### 3.2. Meat Quality Characteristics

The effect of the feeding group on the examined meat quality characteristics was only significant on WB shear force, and the meat of OP lambs was the most tender (Table 5). In addition, 2016-born male and female lambs were more tender than their 2017 counterparts, which was observed because of the significant interaction effect. When the meat quality results were investigated in terms of birth type, the only significant difference between the groups was for express juice, and single-born lambs experienced higher water loss than multiple born lambs when exposed to pressure. A significant gender and the gender × year interaction effect was observed on drip loss, which indicated that male lambs lost more water during the analysis. Male lambs born in 2016 had the highest drip loss percentages, followed by 2017 born males and both years’ female lambs. Additionally, year affected all meat quality traits, except pH_24_, and lambs born in 2017 had higher means in terms of all meat quality characteristics other than drip loss.

In meat colour measurements after 1-h blooming, it was observed that the SG had higher lightness (L*), yellowness (b*), and hue (h*) values than the other groups; however, the difference between the OP in terms of b* value was not significant (Table 6). Single-born lambs had higher redness (a*) and Chroma (C*) values than multiple-born lambs. Additionally, the effects of gender on meat colour were not significant; however, the effect of gender × year interaction was significant and 2016-born males had redder meat than their counterparts (*p* < 0.05). Meat yellowness and hue values were affected from year, concluding with 2017 born lambs having higher values than their counterparts, even though they had lower carcass fatness scores.

## 4. Discussion

The primary objective of this study was to determine the effects of different feeding patterns on the carcass and meat quality of Karacabey Merino lambs slaughtered at the same age. The feeding group affected conformation and fatness scores with omental-mesenteric fat and KKCF percentages of Karacabey Merino lambs. Although SW, CCW, and EBW were similar among the feeding groups, the conformation and fatness scores and OMF percentages of SG lambs were significantly higher than those of both pasture groups. Many studies have reported that concentrate fed lambs had fatter carcasses than pasture lambs because of higher feed intake and lower physical activity [6,26]. Physical activity during grazing causes increased metabolisation of lipid reserves to build muscle tissue, which results in lower carcass fatness, especially subcutaneous fat [10]. Similarly, Borton et al. [4] observed greater carcass weight in concentrate-fed lambs than forage-fed lambs because forage finishing systems increase digestive tract size and decrease external fat. However, there were no significant differences among feeding groups regarding EBW or CCW in the current study. Because of the significant interaction effects, male TP lambs had the lowest conformation scores, while female TP lambs had the lowest fatness scores, indicating that even though the nutritional content between the pasture groups was similar, triticale fed lambs developed less muscle and fat tissue than their counterparts within the same feeding duration.

Feeding did not affect SL and DP significantly; however, as a result of the interactions, the SL of both pasture groups was higher than that of concentrate-fed ones in 2017. Increase in carcass surface area per kilogram live weight and decreased subcutaneous fat thickness resulted in low conformation and fatness scores of pasture groups, lowering the ability of preserving the moisture, which leads to a high shrinkage loss, which is a similar result to those of Smith and Carpenter [27] and Joy et al. [28]. The dressing percentage of single born female lambs was the highest, even though their EBW was lower than the males, their CS, FS, and DP showed that they had more developed carcasses than their counterparts, which is similar to Pérez et al. [29]. There is an order for development of different adipose tissues in carcasses. Fat tissue development gradually increases from mesenteric to subcutaneous (mesenteric, intermuscular, omental, KKCF, and subcutaneous, in that order) [10]. The OMF percentages of pasture groups were similar; however, the lower KKCF percentages of TP lambs indicated that oat pasture lambs had higher energy intakes and had a greater chance of accumulate more fat tissue than TP lambs. As the development of fat tissue begins later than muscle [6,30,31], the TP lambs were not finished their muscle development when compared with their counterparts.

As an expected result, single-born lambs had higher birth weight, EBW, CCW, conformation and fatness scores, and LTMSA, which indicates that they are not only born heavier than their counterparts, but they can also grow more muscles in the same period [32,33,34]. Many studies have reported that single-born lambs grow faster and are heavier than multiple-born lambs, and this is mainly due to having a non-competitive environment during gestation and not sharing milk with a sibling [35,36,37].

Although the male lambs had higher birth weights, the differences between male and female lambs for CCW and LTMSA were not significant. However, female lambs had higher CS and FS, DP, OMF percentage, and KKCF percentages, which indicates that female lambs have fatter carcasses (both subcutaneous and non-carcass fat). As many authors have stated before [35,37,38], oestrogen hormone has a limited effect on both muscle and bone growth; however, it has a supportive effect on fat. In addition, higher fat content (both carcass and non-carcass) in females supports the fact that females can deposit larger amounts of fat at an earlier period of growth, as they mature faster and fatten faster than males [39,40,41,42].

Additionally, the birth year also affected many carcass characteristics. The main reason for this was that the yield in both pastures was low in 2017, which prolonged the fattening period (mean slaughter age was 130.09 in 2017 over 117.89 days in 2016), and resulted with lower slaughter weight, conformation, and fatness scores than 2016-born lambs.

When the carcass joints are commercially classified, the loin rib, hind limb, and anterior ribs are the first category cuts, the shoulder/thoracic limb is secondary, and the flank and neck are the third [10]. Shoulder, hind limb, and the ribs are the early developed carcass joints, as they are the primary body parts for the survival of the offspring. The feeding system affected the shoulder, flank, anterior rib, and tail percentages of Karacabey Merino lambs. TP lambs had higher shoulder percentages, whereas OP lambs had higher anterior ribs percentages than their counterparts. On the other hand, SG lambs developed more of the flank and tail, which are the least favourable carcass joints. Results indicate that OP and TP feeding systems contributed to the growth of first and second-category cuts, while concentrate feed helped to develop third category carcass joints. Similar to our study, Murphy et al. [43] and Karaca et al. [8], observed higher shoulder percentage and leaner flanks in grazing lambs, than in grazing + concentrate and concentrate-fed ones.

Birth type did not affect the primal joint size in a commercially important way, with multiple-born lambs only having a higher kidney percentage, similar to the findings of Thatcher et al. [44]. However, Jucá et al. [34] reported differences between birth type groups in terms of carcass joint percentages. The differences among the studies might be related to the breed, which has a significant influence on the development of the carcass joints [45,46]. Additionally, when the effect of gender was investigated, male lambs had higher shoulder and kidney percentages, while females had higher loin percentages. Similar to our results, Žgur et al. [47] reported that male lambs had higher neck, chuck, and shoulder percentages, whereas females had higher kidney fat, back, and loin percentages.

The feeding programmes used in the study had no effect on the ultimate pH (ranging between 5.52 to 5.58, within acceptable limits declared by Hedrick et al. [48]), which explains the lack of differences in the investigated meat quality traits among feeding systems [3,5,30,49]. In addition, Ripoll et al. [11] stated that even though four fattening systems with various grazing and concentrate levels were used, the ultimate pH values of lamb carcasses in those systems were similar. Slaughtering the lambs on the same day without transportation under the same pre-slaughter conditions might be the key to this result, as it creates the minimum pre-slaughter stress. However, there are some studies [2,12,50,51] report that the feeding system has a significant effect on ultimate pH.

Express juice is a trait used to measure the amount of water lost under pressure [21], which is usually related to the amount of fat stored in the body [8]. Because adipose tissue tends to store more water than muscle or bone, water-holding capacity will be higher in fattier carcasses and they will lose less water under pressure. Among the meat quality characteristics, a significant birth type effect was detected for only for the express juice, which might be a result of the single lambs having higher slaughter weights. Both Vergara et al. [52] and Ekiz et al. [53] found an increase in the express juice of lambs with higher slaughter weights, which was related to lower carcass fatness, similar to our findings with single born lambs. However, this situation was reported as breed and/or weight-class specific in some cases [54]. In addition, Ekiz et al. [55] stated no significant effect of birth type on the express juice of Kıvırcık lambs, which is controversial with our findings.

It has been observed that females have lower DL and WBSF values; therefore, they have tender and juicier meat. This situation is an expected result of females having fattier carcasses than males due to their higher subcutaneous and internal fat content caused by the oestrogen effect [41,42]. Significant gender influences on meat quality traits were also previously reported by Ruiz de Huidobro et al. [56] and Vergara and Gallego [49]; however, both Olleta et al. [57] and Rodríguez et al. [41] reported no differences in water-holding capacity between male and female lambs and explained this situation with similar pH values observed in male and female lambs.

Oat pasture lambs had tender meat according to WB shear force compared with other groups. This may be due to lambs in oat pastures suckling more milk from their dams, since the ewes in the OP had slightly higher daily milk yield than the TP ewes. Juarez et al. [58] reported that suckling lambs had more tender meat than those fed with both milk and concentrate. However, factors such as carcass weight and movement in the pasture or having higher dry matter intake may also affect meat tenderness [59,60].

The meat of the stall group was lighter than that of both grazing groups at 1-h blooming, which might be a result of them being fattier than the grazing groups, since the adipose tissue allows the penetration of light more than the muscle [61]. Both Priolo et al. [6] and Karaca et al. [8] reported higher lightness in SG lambs than in the grass ones, related to lower pH_24_ values of the SG lambs. However, the pH levels of lambs from different feeding groups were similar in our study. Similar to our findings, Díaz et al. [5] also reported lighter meat colour for SG lambs with similar ultimate pH values between concentrate-fed and pasture-fed groups, which they explained with different levels of physical activity.

Increased physical activity in the pasture can increase pigmentation, which can be observed as a higher a* (redness) value in meat [11,62]. However, the differences among the study groups were not significant with respect to the a* values, similar to the findings of Karaca et al. [8].

Both grazing groups showed lower yellowness (b*) values than concentrate-fed SG, which contradicts both Priolo et al. [6] and Carrasco et al. [63], since it is a more expected result because of the higher physical activity and carotenoids intake [11]. Karaca et al. [8] explained this situation with higher ultimate pH levels of pasture groups, which creates a detrimental effect on meat colour. However, the slaughter weights and pH levels were similar in our study.

The effect of birth type was only significant on a* and C* values; single-born lambs had higher values for both parameters. This was an expected outcome because of the higher EBW, conformation, and fatness scores of single-born ones. However, both Jucá et al. [34] and Greeff et al. [64] reported that birth type had no significant effect on redness as a result of similar pH levels.

Meat colour can be affected by the intermuscular fat content, because of the penetration of the light being easier in adipose tissue [41,61]. Even though the fatness levels of female lambs were higher than male lambs, the meat colour of the gender groups was similar. No gender effect on meat colour was also reported by Rodríguez et al. [41], Santos et al. [65], Sañudo et al. [66], Vergara and Gallego [49], and Vergara et al. [52].

## 5. Conclusions

Results indicated that when Karacabey Merino lambs were slaughtered at similar age and weight, concentrate-fed lambs had better conformation and fatness performances rather than pasture-fed lambs. However, pasture-based feeding systems contributed to the growth of the first and second categories of carcass joints, while concentrate-fed lambs developed more fat and less preferred ones. Additionally, except the traits related to subcutaneous and non-carcass fatness, there was no significant difference between stall feeding and pasture feeding lambs. Furthermore, differences among pasture groups were not significant for almost all characteristics examined in the study, except WBSF, suggesting that oat and triticale are interchangeable feed supplies for Karacabey Merino lambs. In conclusion, a pasture-based feeding system is more suitable for Karacabey Merino than a stall feeding system, if lean and tender meat is preferred. However, it should not be ignored that lambs fed in stalls showed better fattening performance in terms of conformation and fatness during the same period.

## Figures and Tables

**Table 1:** Number of animals used in each group.

	2016			2017		
Feeding Group	Ewe	Lambs Born	Lambs Slaughtered	Ewe	Lambs Born	Lambs Slaughtered
**Stall Group (SG)**	12	18	9	12	15	8
**Triticale Pasture (TP)**	12	15	9	12	14	11
**Oat Pasture (OP)**	12	16	9	12	17	12

**Table 2:** Nutritional content of the pastures consumed by the lambs according to the years of the study.

Pastures	DM, %	CP, %	NDF, %	ADF, %	DOM, %	ME, kcal/kg
Winter Pasture—2016	57.87	11.11	63.29	36.25	63.04	2.079
Triticale Pasture—2016	29.07	12.68	53.64	28.06	70.34	2.584
Oat Pasture—2016	26.77	12.89	52.66	26.73	71.28	2.626
Winter Pasture—2017	71.87	9.20	63.41	38.35	61.66	1.995
Triticale Pasture—2017	31.38	14.06	53.63	34.11	66.09	2.390
Oat Pasture—2017	26.98	15.68	50.57	33.43	66.56	2.413

DM: Dry matter, CP: Crude protein, NDF: Neutral detergent fibre, ADF: Acid detergent fibre, DOM: Digestible organic matter, ME: Metabolisable energy.

**Table 4:** Percentages of carcass joints according to feeding group, birth type, gender, and year.

	n	Shoulder, %	Flank, %	Neck, %	Anterior Rrib, %	Loin, %	Hind Limb, %	Tail, %	Kidney, %
**Group**									
Stall	17	18.87 ^b^ ± 0.48	13.41 ^a^ ± 0.35	6.11 ± 0.24	16.47 ^b^ ± 0.39	8.24 ± 0.19	34.56 ± 0.54	0.53 ^a^ ± 0.03	0.60 ± 0.02
Triticale	20	20.69 ^a^ ± 0.45	12.07 ^b^ ± 0.32	6.29 ± 0.23	15.90 ^b^ ± 0.36	8.35 ± 0.18	34.95 ± 0.50	0.42 ^b^ ± 0.03	0.61 ± 0.02
Oat	21	19.15 ^b^ ± 0.44	12.69 ^ab^ ± 0.32	5.71 ± 0.22	17.62 ^a^ ± 0.35	8.19 ± 0.17	34.44 ± 0.49	0.43 ^b^ ± 0.03	0.63 ± 0.02
**Birth Type**									
Single	31	19.76 ± 0.36	12.86 ± 0.26	5.94 ± 0.18	16.46 ± 0.29	8.30 ± 0.14	34.52 ± 0.41	0.48 ± 0.02	0.59 ± 0.01
Multiple	27	19.38 ± 0.39	12.58 ± 0.28	6.13 ± 0.20	16.87 ± 0.31	8.21 ± 0.15	34.78 ± 0.44	0.44 ± 0.02	0.64 ± 0.02
**Gender**									
Male	25	20.24 ± 0.41	12.41 ± 0.29	6.04 ± 0.21	16.64 ± 0.33	8.04 ± 0.16	34.79 ± 0.46	0.43 ± 0.02	0.64 ± 0.02
Female	33	18.90 ± 0.35	13.03 ± 0.25	6.03 ± 0.17	16.69 ± 0.28	8.48 ± 0.14	34.50 ± 0.39	0.49 ± 0.02	0.59 ± 0.01
**Year**									
2016	27	18.70 ± 0.40	13.21 ± 0.29	6.69 ± 0.20	16.23 ± 0.32	8.46 ± 0.16	34.52 ± 0.44	0.51 ± 0.02	0.58 ± 0.02
2017	31	20.44 ± 0.36	12.23 ± 0.26	5.38 ± 0.18	17.10 ± 0.29	8.05 ± 0.14	37.77 ± 0.40	0.41 ± 0.02	0.65 ± 0.01
**Overall mean**		19.57 ± 0.26	12.72 ± 0.19	6.04 ± 0.13	16.66 ± 0.21	8.26 ± 0.10	34.65 ± 0.29	0.46 ± 0.02	0.61 ± 0.01
**Significance**									
Group		**0.013**	**0.025**	0.179	**0.004**	0.806	0.740	**0.013**	0.638
Birth Type		0.474	0.472	0.487	0.344	0.655	0.660	0.188	**0.011**
Gender		**0.017**	0.116	0.954	0.914	**0.042**	0.634	0.066	**0.046**
Year		**0.002**	**0.014**	**<0.001**	0.051	0.058	0.168	**0.002**	**<0.001**

The results in the table are presented as least square means ± standard errors. ^a,b^: The difference between the mean values expressed with different letters in the same column for the feeding groups is statistically significant. n: Number of animals in a group.

**Table 5:** Meat quality traits of lambs according to feeding group, birth type, gender, and year.

	n	pH_0_	pH_24_	Drip Loss, %	Express Juice, %	Cooking Loss, %	WBSF, N
**Group**							
Stall	17	6.26 ± 0.06	5.52 ± 0.03	1.68 ± 0.10	11.69 ± 0.36	32.62 ± 0.48	33.03 ^a^ ± 1.42
Triticale	20	6.31 ± 0.06	5.58 ± 0.02	1.57 ± 0.10	11.51 ± 0.34	32.35 ± 0.44	32.08 ^a^ ± 1.33
Oat	21	6.42 ± 0.06	5.53 ± 0.02	1.71 ± 0.10	10.93 ± 0.33	31.61 ± 0.45	28.05 ^b^ ± 1.33
**Birth Type**							
Single	31	6.38 ± 0.05	5.54 ± 0.02	1.68 ± 0.08	11.88 ± 0.27	32.20 ± 0.37	30.24 ± 1.16
Multiple	27	6.28 ± 0.05	5.56 ± 0.02	1.64 ± 0.08	10.87 ± 0.29	32.19 ± 0.38	31.86 ± 1.15
**Gender**							
Male	25	6.35 ± 0.05	5.56 ± 0.02	1.79 ± 0.09	11.56 ± 0.31	32.65 ± 0.41	32.65 ± 1.24
Female	33	6.31 ± 0.04	5.53 ± 0.02	1.52 ± 0.07	11.29 ± 0.26	31.74 ± 0.35	29.46 ± 1.02
**Year**							
2016	27	6.20 ± 0.05	5.55 ± 0.02	2.20 ± 0.09	9.62 ± 0.30	30.97 ± 0.40	25.76 ± 1.22
2017	31	6.46 ± 0.04	5.54 ± 0.02	1.12 ± 0.08	13.13 ± 0.27	33.42 ± 0.35	36.35 ± 1.05
**Overall mean**		6.33 ± 0.3	5.54 ± 0.01	1.66 ± 0.06	11.38 ± 0.20	32.19 ± 0.27	31.05 ± 0.80
**Significance**							
Group		0.113	0.127	0.526	0.277	0.280	**0.027**
Birth Type		0.145	0.944	0.719	**0.016**	0.990	0.333
Gender		0.610	0.336	**0.020**	0.690	0.100	0.053
Year		**<0.001**	0.865	**<0.001**	**<0.001**	**<0.001**	**<0.001**
GE×Y				**0.026**			**0.011**
GR×BT						**0.017**	

The results in the table are presented as least square means ± standard errors. GR: Group, BT: Birth type, GE: Gender, Y: Year, WBSF: Warner–Bratzler Shear Force. ^a,b^: The difference between the mean values expressed with different letters in the same column for the feeding groups is statistically significant. n: Number of animals in a group.

**Table 6:** Meat colour characteristics of lambs according to feeding group, birth type, gender, and year.

	n	L*	a*	b*	C*	h*
**Group**						
Stall	17	41.51 ^a^ ± 0.55	19.53 ± 0.35	6.67 ^a^ ± 0.32	20.67 ± 0.40	18.90 ^a^ ± 0.77
Triticale	20	39.04 ^b^ ± 0.51	19.18 ± 0.33	5.08 ^b^ ± 0.30	19.90 ± 0.37	14.77 ^b^ ± 0.74
Oat	21	39.39 ^b^ ± 0.50	19.92 ± 0.33	5.84 ^ab^ ± 0.30	20.83 ± 0.37	16.28 ^b^ ± 0.70
**Birth Type**						
Single	31	40.18 ± 0.41	20.04 ± 0.29	6.20 ± 0.24	21.06 ± 0.32	17.27 ± 0.58
Multiple	27	39.78 ± 0.44	19.05 ± 0.28	5.53 ± 0.26	19.87 ± 0.32	16.03 ± 0.62
**Gender**						
Male	25	40.34 ± 0.46	19.50 ± 0.31	6.05 ± 0.27	20.50 ± 0.34	17.24 ± 0.65
Female	33	39.62 ± 0.39	19.58 ± 0.25	5.67 ± 0.23	20.44 ± 0.28	16.06 ± 0.55
**Year**						
2016	27	39.59 ± 0.45	19.69 ± 0.30	6.40 ± 0.27	20.79 ± 0.34	18.02 ± 0.63
2017	31	40.38 ± 0.41	19.39 ± 0.26	5.33 ± 0.24	20.15 ± 0.29	15.27 ± 0.57
**Overall mean**		39.98 ± 0.30	19.54 ± 0.20	5.86 ± 0.18	20.47 ± 0.22	16.65 ± 0.42
**Significance**						
Group		**0.004**	0.273	**0.003**	0.151	**0.001**
Birth Type		0.512	**0.020**	0.068	**0.012**	0.156
Gender		0.252	0.848	0.303	0.893	0.177
Year		0.205	0.454	**0.005**	0.158	**0.002**
GE×Y			**0.019**		**0.016**	

The results in the table are presented as least square means ± standard errors. ^a,b^: The difference between the mean values expressed with different letters in the same column for the feeding groups is statistically significant. GE: Gender, Y: Year. L*: Lightness, a*: redness, b*: yellowness, C*: Chroma, h*: hue angle. n: Number of animals in a group.

## Data Availability

The data presented in this study are available upon reasonable request from the corresponding authors.
